# Downregulation of *DROSHA*: Could It Affect miRNA Biogenesis in Endometriotic Menstrual Blood Mesenchymal Stem Cells?

**DOI:** 10.3390/ijms24065963

**Published:** 2023-03-22

**Authors:** Ana Clara Lagazzi Cressoni, Letícia B. C. Penariol, Cristiana Carolina Padovan, Maristela D. Orellana, Júlio Cesar Rosa-e-Silva, Omero Benedicto Poli-Neto, Rui Alberto Ferriani, Cláudia Cristina Paro de Paz, Juliana Meola

**Affiliations:** 1 Department of Gynecology and Obstetrics, Ribeirao Preto Medical School, University of Sao Paulo, Ribeirão Preto, São Paulo 14049-900, Brazil; 2 Regional Blood Center, Medical School of Hemocenter Foundation of Ribeirão Preto, University of Sao Paulo, Ribeirão Preto, São Paulo 14051-140, Brazil; 3 National Institute of Hormones and Women’s Health (Hormona)—CNPq, Porto Alegre 90035-003, Brazil; 4 Department of Genetics, Ribeirao Preto Medical School, University of Sao Paulo, Ribeirão Preto, São Paulo 14049-900, Brazil

**Keywords:** endometriosis, *DROSHA*, miRNA biosynthesis, MenSC, RT-qPCR

## Abstract

Menstrual blood mesenchymal stem cells (MenSCs) have gained prominence in the endometriosis scientific community, given their multifunctional roles in regenerative medicine as a noninvasive source for future clinical applications. In addition, changes in post-transcriptional regulation via miRNAs have been explored in endometriotic MenSCs with a role in modulating proliferation, angiogenesis, differentiation, stemness, self-renewal, and the mesenchymal–epithelial transition process. In this sense, homeostasis of the miRNA biosynthesis pathway is essential for several cellular processes and is related to the self-renewal and differentiation of progenitor cells. However, no studies have investigated the miRNA biogenesis pathway in endometriotic MenSCs. In this study, we profiled the expression of eight central genes for the miRNA biosynthesis pathway under experimental conditions involving a two-dimensional culture of MenSCs obtained from healthy women (*n* = 10) and women with endometriosis (*n* = 10) using RT-qPCR and reported a two-fold decrease in *DROSHA* expression in the disease. In addition, miR-128-3p, miR-27a-3p, miR-27b-3p, miR-181a-5p, miR-181b-5p, miR-452-3p, miR-216a-5p, miR-216b-5p, and miR-93-5p, which have been associated with endometriosis, were identified through in silico analyses as negative regulators of *DROSHA*. Because DROSHA is essential for miRNA maturation, our findings may justify the identification of different profiles of miRNAs with DROSHA-dependent biogenesis in endometriosis.

## 1. Introduction

Endometriosis is a benign gynecological, estrogen-dependent disease that affects over 10% of women of reproductive age [1]. Its etiopathogenesis is multifactorial and results from aberrant endocrine, immunological, genetic, inflammatory, and angiogenic processes [2]. One of the theories to explain its origin is retrograde menstruation [3], which assumes that menstrual blood reflux carries portions of viable endometrial cells with adhesion and invasion ability to the peritoneal cavity, resulting in ectopic lesions [4,5]. However, their origin is complex and remains undefined [4]. 

In this context, a possible role of mesenchymal stem cells in the endometrium (eMSCs) [6,7], in the menstrual blood (MenSCs) [8], and in endometriotic lesions [9] has been suggested as a player in the establishment and progression of ectopic tissue [10]. MenSCs have recently been the subject of studies on the etiology of endometriosis owing to their high proliferation rates, immunomodulatory capacity, migration to inflammatory niches, clinical applications, and roles in regenerative medicine with no ethical dilemma [11,12]. In addition, given the important particularities evidenced in the endometrium of women with endometriosis [13,14], and although the participation of the endometrium as a carrier of changes is not fully understood, tracking molecular alterations in these progenitor cell types could demonstrate their involvement in the disease. The few studies on differential expression of genes or proteins in endometriotic MenSCs suggest that primary cell alterations are related to impaired decidualization potential and the chronic inflammatory endometrial microenvironment [15,16,17,18].

Gene expression is an intricate mechanism that involves the modulation of transcriptional, post-transcriptional, translational, and post-translational modifications. A discrepancy between the transcript levels and the corresponding proteins observed in endometriosis may be associated with the pathophysiology of the disease and post-transcriptional regulatory mechanisms [19,20,21]. MiRNAs, although not the only ones, constitute one of the main elements responsible for post-transcriptional regulation, having been widely explored in the disease [22,23,24]. In the endometriosis mesenchymal stem cells scenario, the miRNAs described have a role in modulating proliferation, angiogenesis, differentiation, stemness, self-renewal, and mesenchymal–epithelial transition processes [25,26,27,28]. Given the diversity of functions associated with this disease, it is understandable that these molecules are targets of interest in the endometriosis scientific community.

MicroRNAs (or miRNAs) are single-stranded structures 20–30 nucleotides in length. Homeostasis of the miRNA biosynthetic pathway is important for several cellular processes and is related to the self-renewal and differentiation of progenitor cells [29,30,31]. The miRNA biogenesis pathways are classified as canonical and non-canonical, with different combinations of proteins involved in the non-canonical and canonical pathways, mainly DROSHA, DICER, XPO5, and AGO2 [32]. Canonical biogenesis starts with RNA Polymerase II transcription of the miRNA gene [33]. The first long hairpin transcript structure, pri-miRNA, is converted to pre-miRNA by the nuclear microprocessor composed of the DROSHA enzyme and the DGCR8 cofactor [34], and XPO5 (EXPORTIN) mediates the export of pre-miRNA to the cytoplasm [33]. DICER is responsible for cleavage on a loop basis, releasing a small double-stranded RNA carried by an ARGONAUTE protein (AGO), which forms the RNA-induced silencing complex (RISC) [33]. The RISC is responsible for connecting the leading strand of the miRNA to the complementary region of the messenger RNA (mRNA), inducing translational repression, deadenylation, and, consequently, a decline in the mRNA [35,36].

No study has investigated the miRNA biogenesis pathway in endometriotic MenSCs. Whether dysregulation of the central genes involved in the canonical and non-canonical miRNA biogenesis pathways may take part in the etiopathogenic process of endometriosis remains unknown. Our goal, therefore, was to evaluate whether the gene expressions of *DROSHA*, *DGCR8*, *XPO5*, *DICER*, and *AGO1* to *AGO4* are differential in MenSCs of women with endometriosis. Furthermore, to explain a possible post-transcriptional mechanism via miRNAs for the dysregulation of these key genes, we performed in silico analyses to predict miRNAs as regulators of differentially expressed genes (DEGs). 

## 2. Results

This case-control study profiled the expression of eight genes central to the miRNA biosynthetic pathway under experimental conditions involving two-dimensional cultures of MenSCs obtained from healthy women (*n*  =  10) and women with endometriosis (*n*  =  10) using RT-qPCR. The clinical characteristics of the women enrolled in this study were previously described by the authors of [28] (pp. 736, table 1). No significant differences were observed between case and control for clinical data: age (mean 36 ± 3.0 and 35 ± 3.5), body mass index (mean 25.18 ± 2.9 and 26.03 ± 2.4), and menstrual flow collection days (mean 2.7 ± 0.8 and 2.7 ± 0.6), respectively. The patients were infertile and complained of pelvic pain. They all had an ultrasound diagnosis suggesting endometrioma in at least one of the ovaries. The MenSC in vitro model was previously established by our group [28,37]. The cells in this study were stored in a biorepository and previously characterized as MSCs using a panel of 23 markers. No significant differences were observed in the percentages of immunophenotypically labeled cells between the two conditions [28] (pp. 736, table 2), with the expression according to the minimum criteria that define multipotent MSCs [38] and the previously described MenSC profiles [11,39,40].

### 2.1. Expression Profiles of DROSHA, DGCR8, XPO5, DICER, and AGO1 to AGO4

All samples used in this study were considered appropriate for the quantification of gene expression using RT-qPCR, as they presented RNA integrity number (RIN) values ranging from 8.10 to 10 (Figure S1 (Supplementary Materials)), compatible with reference values for well-preserved RNA [41].

**Figure 1:**
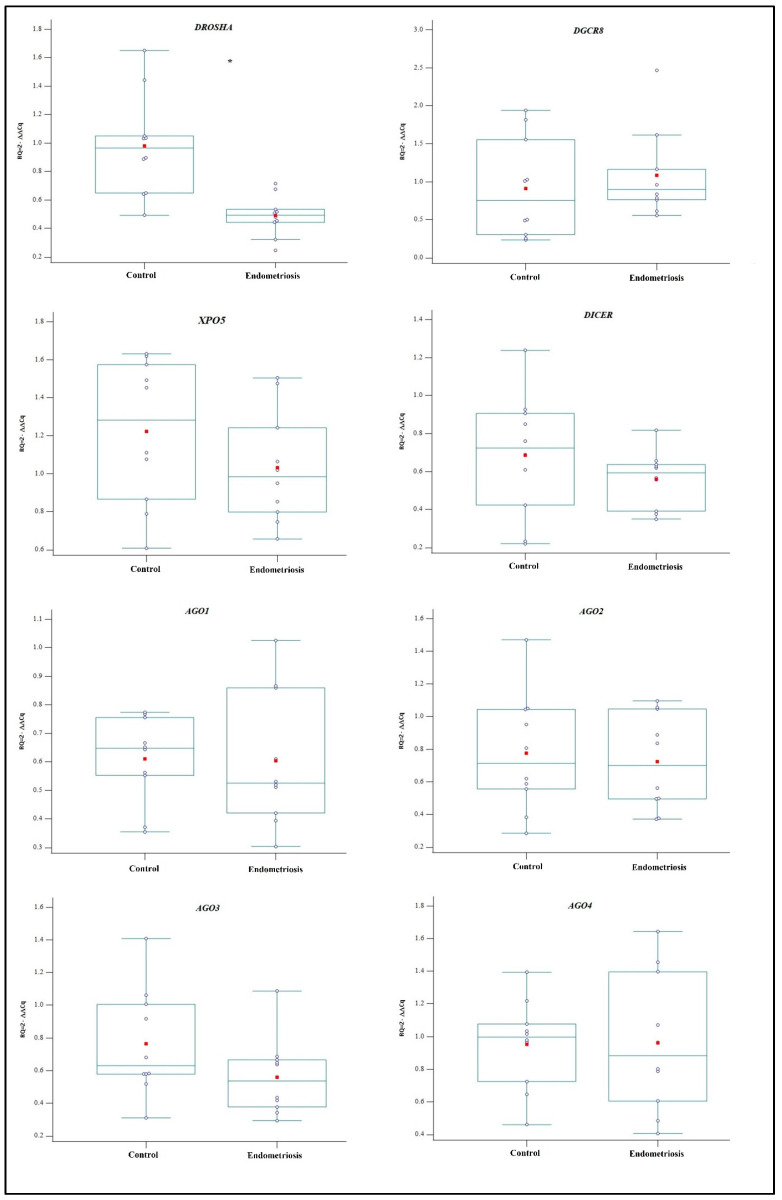
The expression profiles of *DROSHA*, *DGCR8*, *XPO5*, *DICER*, and *AGO1* to *AGO4* genes were obtained in MenSCs from women with and without endometriosis through RT-qPCR. The dots, red markers, and asterisk represent the raw Cq data of each sample, mean value, and significant differences (*p* < 0.05), respectively. Cq = cycle quantification. The box plot was created using MedCalc Statistical Software version 19.5.1 (MedCalc Software Ltd., Ostend, Belgium; https://www.medcalc.org; 2020).

A significant decrease in *DROSHA* of approximately two-fold was found in the endometriosis group (mean 0.48 ± 0.14, *p* = 0.008) compared to that of the control group (mean 0.97 ± 0.35). Although no significant differences in the expressions of the other evaluated genes were observed, these results are inconclusive because only *AGO3* had a test power of 80% (Figure 1).

### 2.2. In Silico Data of miRNA-Downregulated DROSHA

As *DROSHA* is less expressed in endometriosis MenSCs, we selected 17 miRNAs predicted to be negative regulators of *DROSHA* using DIANA-TarBase v8, adopting an interaction prediction score of <0.4 (see search results in https://dianalab.e-ce.uth.gr/html/diana/web/index.php?r=tarbasev8%2Findex&miRNAs%5B0%5D=&genes%5B0%5D=&genes%5B1%5D=DROSHA&species%5B0%5D=1&regulation_types%5B0%5D=DOWN&sources%5B0%5D=1&sources%5B1%5D=7&sources%5B2%5D=9&publication_year=&prediction_score=&sort_field=score&sort_type=DESC&query=1&page=1, accessed on 28 December 2022). In addition, we searched the literature for miRNAs that have already been studied in endometriosis (Table 1).

## 3. Discussion

To our knowledge, this is the first study to evaluate the expression of *DROSHA*, *DGCR8*, *XPO5*, *DICER*, and *AGO1* to *AGO4* in MenSCs from women with endometriosis. Here, we report a two-fold decrease in *DROSHA* expression during the disease. Nine miRNAs (miR-128-3p, miR-27a-3p, miR-27b-3p, miR-181a-5p, miR-181b-5p, miR-452-3p, miR-216a-5p, miR-216b-5p, and miR-93-5p) were experimentally verified as negative regulators of *DROSHA* through in silico analyses and, according to the literature, have been associated with endometriosis. However, the reasons and mechanisms for the *DROSHA* changes in endometriosis and their impact on mesenchymal stem cells in the disease are unknown.

DROSHA is the first enzyme to function in the biosynthesis of miRNAs and is responsible for the cleavage of the hairpin structure in which the mature miRNA is sheltered. *DROSHA* expression, activity, and specificity levels are controlled by autoregulation between DROSHA and DGCR8, post-translational modifications, and the linkage of proteins to the terminal portion of pri-miRNA [33]. Therefore, considering that the efficiency of DROSHA processing is crucial for determining the abundance of miRNAs, our results suggest an impact on the maturation of miRNAs in endometriotic MenSCs, which may contribute to the establishment of endometrial foci and consequently contribute to the etiopathogenesis of the disease. A few studies addressing altered miRNAs in endometrial and menstrual MSCs in endometriosis suggest a role in modulating proliferation, angiogenesis, differentiation, stemness, self-renewal, and the mesenchymal–epithelial transition processes [25,26,27,28]. 

The functions of DROSHA and DICER have been discussed in terms of how their manipulation alters cell plasticity, growth, and division, as well as stem cell differentiation and self-renewal [29,30,57,58,59]. DICER and DROSHA downregulation in mesenchymal stromal cells from myelodysplastic syndrome was associated with a global down expression of microRNAs involved in cell migration and attachment [59]. Global repression of miRNAs increased transformation in human cancer cell lines and promoted prominent cell motility, favoring the establishment of tumors and metastases [58]. We recently reported decreased DROSHA protein levels in adenomyosis and suggested a possible relationship between its pathophysiology and endometrial cancer [60]. Interestingly, DROSHA may regulate cell cycle progression in human MSCs via a miRNA-independent mechanism, potentially by regulating rRNA processing [61]. In light of these reports, we questioned whether deficient *DROSHA* expression in endometriosis MenSCs could affect the maturation of a set of miRNAs that depend on the canonical and non-canonical (DICER-independent) pathways. DICER-independent processing has already been well described for the miR-451a [62], which correlates with the endometriotic tissue survival status [63,64]. Thus, to elucidate the impact of our findings, performing a global analysis of miRNAs and functional tests in endometriotic progenitor cells would be interesting. Considering endometriosis as a multifactorial disorder in which carriers have an altered hormonal and inflammatory environment [5], we may question the impact of these alterations on the MenSCs present in retrograde menstrual flow. This could explain the change in *DROSHA*, the altered biogenesis of miRNAs, and consequently, genes regulated by them in these cells, which may contribute to the viability, implantation, and proliferation of endometriotic foci in the pelvic cavity. Steroids modulate miRNA expression at the transcriptional level and regulate their biosynthesis [64]. Exposure of endometrial epithelial cells to estrogen and progesterone combined alters the expression of *DROSHA*, *DGCR8*, *XPO5*, and *DICER1* [65]. As described by the authors of [66], a physical association occurs between the Estrogen Receptor Alpha (ERα) and the estrogen complex with the microprocessor subunits DROSHA, p68, and p72, which leads to its dissociation into a subset of pri-miRNAs. Given the estrogen-dependent characteristics of endometriosis and progesterone resistance found in many patients [67], the hormonal context of the disease could be involved in the differential modulation of miRNAs in MenSCs from patients with endometriosis. Therefore, future research using appropriate methodologies to evaluate this mechanism would be interesting. 

Furthermore, dysregulation of miRNAs may contribute to the pathogenesis and chronic inflammation in autoimmune and inflammatory diseases [68], such as endometriosis. It has been demonstrated that DROSHA and DICER-dependent miRNA biogenesis are indispensable for immune regulation and the prevention of inflammatory diseases via Foxp3+ regulatory T cells (T reg) [69]. Thus, miRNAs may play an essential role in the inflammatory context of illness [70]. It is possible that this microenvironment contributes to the reduction in *DROSHA* expression in MenSCs, given that alterations in DROSHA are related to inflammatory processes, as previously described, and that the eutopic endometrium of women with endometriosis III and IV presents a sustained stress profile due to the enrichment of TGF signaling pathways, interferon alpha/gamma responses, and the prevalence of natural killer T cells [13].

Our group recently published the first study that integrated transcriptome and proteome data in endometriotic MenSCs; of note, we found no major changes in these two different landscapes [17]. Thus, DROSHA compensatory mechanisms may be acting in these progenitor cells, making it favorable to study miRNoma under these experimental conditions to profile the miRNAs in these cells to understand the mechanisms involved in the disease.

To explain a possible post-transcriptional mechanism via miRNAs to reduce *DROSHA* in our study, we selected miRNAs predicted to be negative regulators of *DROSHA* using the DIANA-TarBase database. Furthermore, we linked these miRNAs to disease (Table 1). The miRNAs miR-128-3p, miR-27a-3p, miR-27b-3p, miR-181a-5p, miR-181b-5p, miR-452-3p, miR-216a-5p, miR-216b-5p, and miR-93-5p are targets for future functional studies as they are related in endometriosis to diagnostic biomarkers [42], autophagy [43], and the regulation of inflammation, vascularization, and angiogenesis of the endometrium during the menstrual cycle and decidualization [44]; fibrosis modulation [45]; enhancement of cell proliferation, migration, and invasion [46]; expression influenced by E2 and BPA [44]; regulation of human endometrial stromal cell decidualization [55]; expression regulated by the menstrual cycle [42]; stromal cell invasion and migration [48]; expression exclusively in ectopic stromal cells [48]; and sustained cell proliferation, cellular adhesion, and apoptosis [52]. 

The strength of our study is that we used stringent inclusion criteria to define the biological groups as homogeneously as possible. Nevertheless, our small sample size did not allow the detection of significant differences in the expression of *DGCR8*, *XPO5*, *DICER*, *AGO1*, *AGO 2*, and *AGO4*. In addition, we applied the gold standard technique for gene expression quantification, RT-qPCR [71]. Despite these advantages, the main limitation of this study is that our experimental design did not allow us to prove the impact of dysregulation of miRNA biosynthesis in MenSCs, requiring future studies to evaluate the global expression of miRNAs in these cells. Another area for improvement is analysis after cell culture, which can mask the cellular environment and its molecular regulation, making it difficult to surpass the same results for in vivo systems [37,67]. However, in vitro studies are still the best choice for research in this field.

## 4. Materials and Methods

### 4.1. Ethics Statement, Settings, and Duration 

This case-control study was performed from November 2019 to December 2020 under the approval of the Research Ethics Committee of the University Hospital of the Ribeirao Preto Medical School (HCRP 3644/2019). The MenSCs used in this study were collected from November 2014 to December 2016 (HCRP 15227/2012), following the ethics guidelines established by the Declaration of Helsinki. They were transferred to a biorepository (HCRP 3644/2019) in the Human Reproduction Sector of the Department of Gynecology and Obstetrics of the Ribeirão Preto Medical School. All participants involved in the study signed an informed consent form.

The women were recruited from the Assisted Reproduction Program of the University Hospital of the Ribeirao Preto Medical School and the Reference Center for Women’s Health in Ribeirao Preto (MATER). The in vitro model was established from November 2014 to December 2016 in partnership with the Regional Blood Center Medical School of the Hemotherapy Center of Ribeirão Preto, University of São Paulo. RT-qPCR was performed at the Multiuser Molecular Biology Laboratory of the Department of Gynecology and Obstetrics of Ribeirão Preto Medical School of the University of São Paulo.

### 4.2. Participants and Eligibility Criteria

The recruited women were aged 18–40 y old, with eumenorrheic menstrual cycles (intervals from 24 to 32 d ± 3 d; 2 to 7 d of duration), without hormone therapy for at least three months before the collection, and no uterine disorder, systemic disease, tumor, endocrinopathy, or cardiovascular or rheumatological diseases. In the case (endometriosis) group, ten women with a histological and laparoscopic diagnosis of endometriosis were classified as III or IV according to the criteria defined by [72]. We selected patients who had undergone surgical treatment for an average of 6 y (SD ± 3.7) before collection. Stem cells have a tropism for endometriotic lesions [73]. Thus, we selected patients who had presented diagnostic imaging findings suggestive of endometrioma at the time of collection as evidence of active disease in the pelvic cavity. For the control group, 10 fertile patients (with children and no history of recurrent abortion) were selected. These patients had no clinical symptoms of endometriosis or endometriotic lesions, as evidenced via laparoscopy during tubal ligation, which occurred for up to one year before collection.

### 4.3. Sample Collection, Isolation of MenSCs through 2D Cultivation, and Characterization of Cells Stored in the Biorepository

Menstrual blood was collected using a silicone menstrual cup (InCiclo, São Paulo, Brazil) previously sterilized with gamma radiation and inserted into the vagina for 3 h during the second, third, or fourth day of the menstrual cycle. Menstrual blood samples were transferred to a solution containing 1X phosphate-buffered saline (PBS) (#10010023; Thermo Fisher Scientific, Waltham, MA, USA), 10X antibiotic–antimycotic solution (#15240-062; Gibco, Grand Island, NY, USA), and 10% acid citrate dextrose solution (JP Farma, São Paulo, Brazil). The samples were stored at 4 °C for up to 4 h. 

Human MSCs from menstrual blood were obtained according to the protocol previously reported by the authors of [8]. The mononuclear cell layer was isolated via density gradient centrifugation at 800× *g* for 30 min at 22 °C using Ficoll-Hypaque (#71-7167-00AG; GE Healthcare Bio-Sciences, Uppsala, Sweden). The cell interface was transferred to a new tube and washed twice with a mixture of 1X PBS, 3X antibiotic–antimycotic solution, and 10% acid citrate-dextrose solution, followed by centrifugation at 450× *g* for 10 min at 22 °C. After washing, the cells were cultured in α-minimum essential medium (α-MEM) (#11900-016, 5) supplemented with 15% fetal bovine serum (#SH30071.03, –HyClone; GE Healthcare Life Sciences, Logan, UT, USA), 1% penicillin/streptomycin (#15140-122; Gibco), 10 mM HEPES (#H4034; Merck Millipore, Billerica, MA, USA), and 20 mM sodium bicarbonate (#56297; Merck) at 37 °C with 5% CO_2_ and 85% humidity. The culture medium was changed every 2–3 d until the adherent cells reached 80–90% confluence. The cells were sub-cultured using 0.05% trypsin-EDTA solution (#25300054; Gibco) until passage 3 (P3) for cell characterization analysis (early culture).

MenSCs fulfill the minimum criteria for multipotent MSCs as defined by the International Society for Cell Therapy (ISCT) [38]. The cells were immunophenotypically characterized by a panel of 23 markers using a FACSCalibur flow cytometer (BD Biosciences, San Jose, CA, USA) and CellQuest software (version 4.0; BD Biosciences, USA). Following the characterization protocols suggested by the ISCT, MenSCs were differentiated into adipocytes and osteocytes following the protocols and means of inducing differentiation described by the authors of [39]. The results for this characterization and the information on the markers used have been described previously by the authors of [28] (pp. 736 table 2, figures S1 and S2). The cells were expanded until P3 for molecular biology protocols.

### 4.4. Isolation, Integrity, and Quantification of Total RNA and cDNA Synthesis

According to the manufacturer’s instructions, the total RNA was extracted using the Allprep DNA/RNA/miRNA Universal Kit (#80224; Qiagen, Valencia, CA, USA). After treatment of the samples with the Ambion DNA-free Kit DNase Treatment and Removal (#AM1906; Invitrogen, Carlsbad, CA, USA) to remove the DNA contaminants, the integrity of the total RNA was evaluated using the 2100 Bioanalyzer equipment (Agilent Technologies, Palo Alto, CA, USA) and the Agilent RNA 6000 Nano Kit (#5067-1511; Agilent). Only samples with an RNA Integrity Number (RIN) greater than eight were included in the study. The total RNA concentrations were determined using fluorimetry in a Qubit 2.0 Fluorometer (Invitrogen) and the Qubit RNA BR Assay Kit (#Q10210; Invitrogen). The cDNA was synthesized from 1000 ng of the total RNA using a High-Capacity RNA-to-cDNA Kit (#4387406; Applied Biosystems, Foster City, CA, USA) according to the manufacturer’s instructions. The final cDNA product was diluted 1:4 with nuclease-free water and stored at −20 °C until qPCR was performed.

### 4.5. Expression Detected Using RT-qPCR

RT-qPCR was performed using the TaqMan Gene Expression Assays (#4331182, Thermo Fisher) using the hydrolysis probes *DROSHA* (Hs00203008_m1), *DGCR8* (Hs00377897_m1), *XPO5* (Hs00382453_m1), *DICER* (Hs00229023_m1), *AGO1* (Hs00201864_m1), *AGO2* (Hs01085579_m1), *AGO3* (Hs01087121_m1), and *AGO4* (Hs00214142_m1) as targets, and *CASC3* (Hs00201226_m1), *EIF2B1* (Hs00426752_m1), and *POP4* (Hs00198357_m1) as reference genes. These reference genes were chosen because of their stability in MenSCs [37]. The reactions were performed for each sample in triplicate under the following conditions: 5 μL of 2X TaqMan Fast Advanced Master Mix (#4444557; Applied Biosystems), 0.5 μL of 20X TaqMan Gene Expression Assay (#4331182; Thermo Fisher), and 4.5 μL of cDNA diluted 1:4 in a final volume of 10 μL reaction. The PCR conditions were 95 °C for 20 s, followed by 40 cycles of 95 °C for 3 s, and 60 °C for 30 s using the ABI Prisma 7500FAST equipment (Applied Biosystems). We considered the technical replicates acceptable with the maximum difference between the Cq values (cycle quantification) for up to 0.3 cycles. Thermo Fisher Scientific guarantees that the amplification efficiencies of the assays used are close to 100% and states that it is unnecessary to measure the efficiency of the normalization calculations (see application note https://assets.thermofisher.com/TFS-Assets/LSG/Application-Notes/cms_040377.pdf, accessed on 20 January 2023).

The relative quantification (RQ) of gene expression was calculated for each sample according to the 2-ΔΔCT method [74] using the Thermo Fisher Connect Platform (version 4.3; Thermo Fisher, UK) available online (http://www.thermofisher.com/br/en/home/digital-science/thermo-fisher-connect.html, accessed on 20 January 2023). A cDNA pool from all control samples was used as the reference sample in the normalization calculation.

### 4.6. Statistical Analysis

Analyses were performed using SAS Statistical Software (version 9.4; SAS Institute, Inc., Cary, NC, USA). Initially, tests for homogeneity of variances and frequency distribution of residues were performed using histograms and tests in PROC UNIVARIATE (SAS Inc., Cary, NC, USA). Logarithmic transformation of the RQ data was unnecessary, as linearity assumptions were met. The *t*-test for independent samples was applied according to General Linear Model procedures to compare the mean values of RQ (2^−ΔΔCt^) between the control and the endometriosis groups. The average RQ values were used to calculate the fold-change in gene expression in the control group regarding the case group. Significance parameters were α = 0.05, and the power of the test (1-β) was at least 0.8. 

### 4.7. In Silico Analysis for Predicting miRNA Targets

We searched for miRNAs predicted to be regulators of DEGs using DIANA-TarBase v8 (http://www.microrna.gr/tarbase, accessed on 28 December 2022), a reference database dedicated to indexing experimentally supported miRNA targets [75]. Based on the differential expression results, we applied a filter (regulation type: down or up) to select miRNAs with positive or negative target regulations. 

## 5. Conclusions

For the first time, this study has described that endometriotic MenSCs have a two-fold decrease in *DROSHA* transcripts. We also highlighted miRNAs miR-128-3p, miR-27a-3p, miR-27b-3p, miR-181a-5p, miR-181b-5p, miR-452-3p, miR-216a-5p, miR-216b-5p, and miR-93-5p as targets for future functional studies on DROSHA mechanisms in MenSCs. Because DROSHA is part of the multiprocessor complex and is essential for the nuclear processing of pri-miRNA maturation, our findings may justify the identification of different profiles of miRNAs due to compromised DROSHA-dependent biogenesis in endometriosis. Furthermore, our results are fundamental for identifying future research challenges and opportunities and leveraging knowledge about mesenchymal stem cells as players in endometriosis etiopathogenesis.

## Figures and Tables

**Table 1:** MiRNAs that negatively regulate DROSHA were identified through an *in silico* analysis and reported in the literature as associated with endometriosis.

miRNA	DianaTools—TarBase v.8 Prediction Score	Endometriosis Literature		
		Expression Level	Sample	Reference
hsa-miR-30b-3p	0.945			No study
hsa-miR-149-5p	0.743			No study
hsa-miR-128-3p	0.739	↑	Plasma of endometriosis samples compared to control samples	[42]
hsa-miR-27a-3p	0.653	↓	Patient plasma-derived extracellular vesicles compared with healthy, fertile control	[43]
hsa-miR-27b-3p	0.652	↓	Human endometrial stromal cells exposed to estradiol	[44]
		↑	Endometriosis human endometrial stromal cells (HESCs) compared with controls	[45]
		↑	Human endometriosis cell line hEM15A	[46]
hsa-miR-181b-5p	0.558	↑	Human endometrial stromal cells exposed to estradiol	[44]
		↓	Endometriosis endometrial stromal cells treated with Saponin extract	[47]
hsa-miR-181d-5p	0.557			No study
hsa-miR-452-3p	0.556	↓	Endometriosis plasma during the luteal phase of the menstrual cycle	[42]
hsa-miR-216b-5p	0.538	↑	Ectopic stromal cells compared to eutopic stromal cells	[48]
hsa-miR-216a-5p	0.526	↓	Endometriosis eutopic endometrial stromal cells	[49]
		↓	Eutopic endometrium compared with ectopic endometrium.	[50]
hsa-miR-676-3p	0.525			No study
hsa-miR-93-5p	0.519	↑	Endometriomas compared with ovarian cancer samples	[51]
		↓	Superficial endometriosis compared with deep endometriosis and endometriomas	[52]
		↑	Endometrial mesenchymal stromal cells treated with endometriotic serum	[53]
		↑	Endometrioma compared with eutopic endometrium	[54]
hsa-miR-181a-5p	0.502	↑	Human endometrial stromal cells treated with 8-bromoadenosine-cAMP and medroxyprogesterone acetate	[55]
		↑	Endometriotic cyst stromal cells compared with normal endometrial stromal cells	[56]
hsa-miR-181c-5p	0.502			No study
hsa-miR-32-5p	0.495			No study
hsa-miR-92b-3p	0.480			No study
hsa-miR-876-3p	0.480			No study

## Data Availability

Raw RT-qPCR data are available upon request from the corresponding author.

## Supplementary Materials

The supporting information can be downloaded at: https://www.mdpi.com/article/10.3390/ijms24065963/s1.
