# Supplementary material for: Downregulation of DROSHA: Could It Affect miRNA Biogenesis in Endometriotic Menstrual Blood Mesenchymal Stem Cells?
Source: Int J Mol Sci. 2023 Mar 22;24(6):5963. doi: 10.3390/ijms24065963 (PMC10057010; doi:10.3390/ijms24065963)
Supplement: Supplementary file 1 [file ijms-24-05963-s001.zip › ijms-2205243-supplementary.pdf]

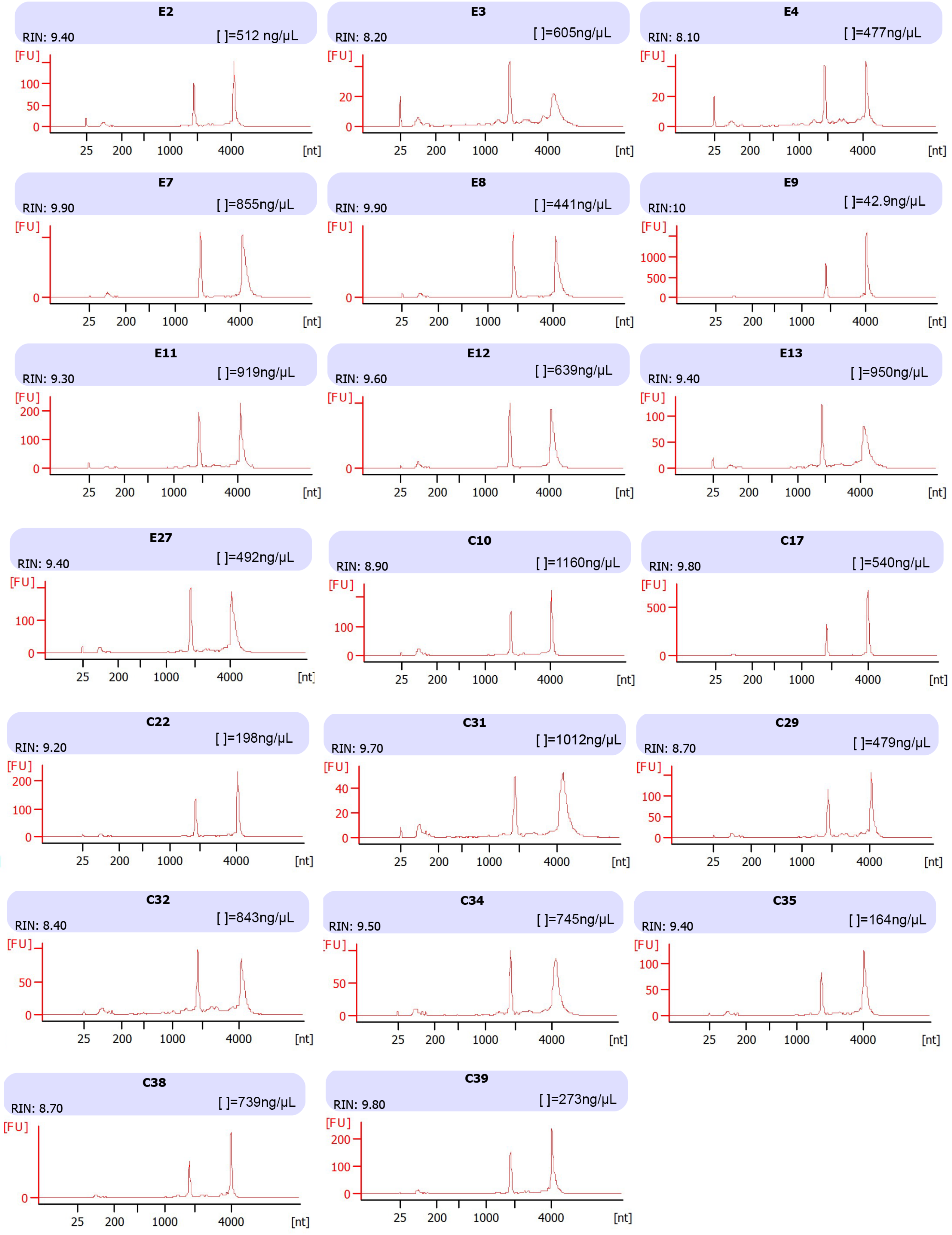

Figure S1: Electropherograms showing the integrity of the total RNA of each sample. The RNA integrity number (RIN) was performed using Agilent 2100 Expert B.02.07.SI532 software. The letters E and C represent, respectively, endometriosis and control. [ ] = RNA concentration obtained by the Qubit 2.0 fluorometer. The two largest peaks represent 18S and 28SrRNA, respectively.
